# Transcriptome-Wide Identification and Expression Analysis of Genes Encoding Defense-Related Peptides of *Filipendula ulmaria* in Response to *Bipolaris sorokiniana* Infection

**DOI:** 10.3390/jof10040258

**Published:** 2024-03-28

**Authors:** Ekaterina A. Istomina, Tatyana V. Korostyleva, Alexey S. Kovtun, Marina P. Slezina, Tatyana I. Odintsova

**Affiliations:** 1 Laboratory of Molecular-Genetic Bases of Plant Immunity, Vavilov Institute of General Genetics RAS, 119333 Moscow, Russia; mer06@yandex.ru (E.A.I.); tatkor@vigg.ru (T.V.K.); omey@list.ru (M.P.S.); 2 Laboratory of Bacterial Genetics, Vavilov Institute of General Genetics RAS, 119333 Moscow, Russia; kovtunas25@gmail.com

**Keywords:** plant immunity, fungal pathogens, antimicrobial peptides, signaling peptides, high-throughput transcriptome sequencing (RNA-seq), *Filipendula ulmaria* (L.) Maxim., *Bipolaris sorokiniana*

## Abstract

Peptides play an essential role in plant development and immunity. *Filipendula ulmaria,* belonging to the Rosaceae family, is a medicinal plant which exhibits valuable pharmacological properties. *F. ulmaria* extracts in vitro inhibit the growth of a variety of plant and human pathogens. The role of peptides in defense against pathogens in *F. ulmaria* remains unknown. The objective of this study was to explore the repertoire of antimicrobial (AMPs) and defense-related signaling peptide genes expressed by *F. ulmaria* in response to infection with *Bipolaris sorokiniana* using RNA-seq. Transcriptomes of healthy and infected plants at two time points were sequenced on the Illumina HiSeq500 platform and de novo assembled. A total of 84 peptide genes encoding novel putative AMPs and signaling peptides were predicted in *F. ulmaria* transcriptomes. They belong to known, as well as new, peptide families. Transcriptional profiling in response to infection disclosed complex expression patterns of peptide genes and identified both up- and down-regulated genes in each family. Among the differentially expressed genes, the vast majority were down-regulated, suggesting suppression of the immune response by the fungus. The expression of 13 peptide genes was up-regulated, indicating their possible involvement in triggering defense response. After functional studies, the encoded peptides can be used in the development of novel biofungicides and resistance inducers.

## 1. Introduction

Supplying sufficient food for the growing world population is of prime importance for mankind. Diseases caused by pathogens and pests pose the greatest challenge to cultivated crops. The total losses of agricultural products due to pathogenic fungi, oomycetes, bacteria, nematodes, insect pests, and viruses can reach 11–30% on a global scale [1]. The greatest damage is caused by pathogenic fungi [2,3]. Since the middle of the 20th century, the severity and scale of fungal diseases have been constantly increasing [4,5]. The estimated pre-harvest and post-harvest losses amount to 10–23% [4].

To control diseases, various strategies are used in agriculture. One of the most potent of them is chemical control, which includes the use of fungicides which considerably decrease crop losses [6]. Although fungicides prevent the spread of infection, their use is unsafe since they affect beneficial soil microbiota, accumulate in food chains disturbing the ecological balance, and promote the appearance of resistant strains of pathogens. The employment of resistant plant varieties carrying resistance genes is another strategy for controlling the infection. However, resistance is rarely long-lasting and sooner or later new resistant races emerge [7]. The alternative strategy is the use of non-pathogenic strains that provide biocontrol and thereby reduce the incidence of disease. However, the effectiveness of the protection provided by these biological agents is highly dependent on various factors, such as geographical and climatic conditions, among others.

Plants protect themselves from pathogens and pests by producing a plethora of antimicrobial compounds including secondary metabolites, phytoanticipins and phytoalexins, antimicrobial proteins (>10 kDa) and peptides (AMPs) (<10 kDa). AMPs represent an essential part of the innate immunity system of all multicellular organisms [8]. In accordance with 3D structure similarity, AMPs are subdivided into several families: defensins, non-specific lipid-transfer proteins (nsLTPs), thionins, hevein- and knottin-like peptides, cyclotides, α-hairpinins, etc. AMPs display broad-spectrum inhibitory activity against diverse pathogens: bacteria, fungi, and viruses [9,10,11]. AMPs are effective against both plant and human pathogens, including multidrug-resistant strains, by targeting plasma membranes and/or intracellular components of pathogens [12]. They are not toxic to mammalian cells [9]. Furthermore, in contrast to conventional antibiotics, they slowly select resistant pathogens [13].

Since plant AMPs are natural, nontoxic, rapidly acting, and highly efficient antimicrobials with low incidence of resistance development in pathogens, they show great promise for the production of disease-resistant crops. Their potential has been demonstrated by the heterologous expression of AMP genes in transgenic plants. Numerous studies have shown that overexpression of AMP genes in model and crop plants results in enhanced resistance to pathogens. These encouraging results were obtained for various AMP families: defensins [14,15,16,17,18,19], thionins [20,21,22], hevein-like peptides [23,24,25], snakins [26,27,28], nsLTPs [29,30,31], etc.

For example, overexpression of a wasabi defensin in transgenic rice increased resistance to the fungus *Magmasporthe grisea* [14]. Petunia floral defensins enhanced resistance to *Fusarium* wilt in transgenic banana plants [18]. Overexpression of a *snakin-1* gene in transgenic potato plants enhanced resistance to *Rhizoctonia solani* and *Erwinia carotovora* [26]. Overexpression of an nsLTP antimicrobial protein gene from *Leonurus japonicas* in transgenic *Populus tomentosa* increased resistance to the fungal pathogens *Alternaria alternata* and *Colletotrichum gloeosporioides* [31]. Transgenic plants of *Nicotiana benthamiana* overexpressing *LTP1* showed enhanced resistance to the tobacco mosaic virus [29]. In addition to the production of transgenic plants, in vitro application of AMPs can be also an effective way to control plant diseases [32].

An obvious advantage of AMPs is that they rapidly kill pathogens at all developmental stages including spores, while most agricultural antibiotics and fungicides act only on pathogens in the growing stage [32]. Other important merits of AMPs essential for plant protection include synergism with antimicrobials and fungicides and promotion of plant growth by modulating symbiotic microflora [32].

Another group of plant biologically active peptides showing promise for plant protection include signaling peptides, which regulate immune response in plants [33]. This group is referred to as “phytocytokines” [34,35]. They protect plants against diseases by triggering the immunity mechanisms. For example, treatment of plants with the elicitor Pep peptides enhances their resistance to fungal and bacterial pathogens and herbivores. *Zea mays* plants treated with the elicitor peptide ZmPep3 showed enhanced resistance to herbivores due to the induced production of phytoalexins and volatiles [36]. Pretreatment of peach plants with *Prunus persica* peptides PpPep1 and PpPep2 protect them from *Xanthomoas asboricola* pv. *pruni* infection [37].

As natural antibiotics and immune response regulators, AMPs and signaling peptides represent a valuable, but still insufficiently explored, pool of novel anti-infective agents and regulatory molecules which can be used in the development of new antimicrobials and environmentally friendly plant protection agents, the need for which increases every year [32].

Fungi belonging to the genus *Bipolaris* cause common root rots and crown rot diseases, which are widespread in different zones of cultivation of cereal crops. In addition to root rot, *Bipolaris sorokiniana* is the causal agent of other destructive diseases, such as spot blotch, seedling blight, head blight, and black point [38]. *Bipolaris* spp. have a wide host range and are able to infect not only cereals, but also plants from other families [39].

Different methods have been used to control the diseases caused by *B. sorokiniana* [38]. The use of the endophytic bacterium *Pseudomonas mediterranea*, the actinobacterium *Nocardiopsis dassonvillei*, the bacterial strain *Lysobacter enzymogenes* C3, and the fungal strain *Rhizoctonia* BNR-8-2 as biological control agents was successful in suppressing common root rot and crown rot [40,41]. Crop rotation is also an important management strategy to control these diseases [42]. Breeding for resistance to spot blotch is the optimal option to control spot blotch. Several fungicides, such as carbendazim, propiconazole, and azoxistrobin have been used with good results in the management of spot botch. Biocontrol agents, together with methyl jasmonate or salicylate, were also found to be effective in controlling this disease [43,44]. Management of black point in seeds can be achieved through the use of resistant varieties, fungicides (tebuconazole, imidacloprid, difenoconazole, and cyproconazole), or biocontrol agents [45]. The limitations of all these methods are the same as for the treatment of all fungal infections mentioned above.

The shortcomings of existing approaches to control diseases caused by *B. sorokiniana* and other fungal pathogens urgently require the development of alternative means of pathogen control. Wild plant species resistant to biotic stress are increasingly attracting the attention of researchers as sources of antimicrobial compounds and resistance modulators. In this study, a wild plant species *Filipendula ulmaria* (L.) Maxim. was selected to identify biologically active peptides involved in the activation and regulation of the immune response to the infection with *B. sorokiniana*.

*F. ulmaria* is a perennial plant of the Rosaceae family. It is used as a leaf vegetable, and the flowers and leaves are used to prepare a healthy tea, rich in biologically active compounds [46]. The plant has long been used in folk medicine and is included in the official pharmacopoeia of many Western European countries. The meadowsweet extracts have anti-septic, hepatoprotective, analgesic, anti-inflammatory, gastroprotective, anti-coagulant, anti-rheumatic, immunomodulatory, and cytotoxic properties, and are used in the treatment of various diseases such as arthritis, rheumatism, colds, and conjunctivitis [47].

In vitro tests have shown that *F. ulmaria* leaf extracts are capable of inhibiting growth of the human pathogen *Helicobacter pilori* [48]. The seed extracts of *F. ulmaria* suppress the growth of the Gram-negative bacterium *Eischerichia coli* O157:H7 causing foodborne infections, as well as a wide range of plant pathogens such as *Fusarium oxysporum* and *B. sorokiniana* [49]. To identify the secondary metabolites responsible for the biological activities of meadowsweet, studies of *F. ulmaria* metabolome were conducted. They resulted in the identification of 119 compounds, of which 69 were specific to meadowsweet [50]. A rich diversity of phenolic constituents was detected. However, their role in the biological activity of *F. ulmaria* remains unexplored. Moreover, biologically active compounds of the polypeptide nature, antimicrobial and defense-related signaling peptides, in this species have not been studied so far. The objective of this study was to explore the repertoire of AMPs and signaling peptides in *F. ulmaria* and their expression profile in response to the infection with *B. sorokiniana* using RNA-seq. RNA-sequencing technology has been widely used to explore host responses to infection with pathogens at a whole-genome level. It provides an integrative view of plant reactions mounted by the attack of a pathogen. In our work, of thousands of genes, we focused on AMP and signaling peptide genes. Using an earlier developed algorithm, we studied the array of peptide genes in *F. ulmaria* and their regulation by *B. sorokiniana* infection. As a result of this study, we discovered as many as 84 novel peptide genes in de novo assembled transcriptomes of *F. ulmaria* and identified differentially expressed genes (DEGs), and discussed their role in response to *F. ulmaria* plants subjected to *B. sorokiniana* infection.

The discovered peptide molecules identified by transcriptome analysis and further functionally characterized can serve as valuable templates for the creation of novel biofungicides and immunity inducers.

## 2. Materials and Methods

### 2.1. Biological Material

Seeds of *Filipendula ulmaria* (L.) Maxim. collected in the Moscow region (Russia) were used in the experiment.

*Bipolaris sorokiniana* strain VKM F-4006 was obtained from the State Collection of Plant Pathogenic Microorganisms at the All-Russian Research Institute of Phytopathology (Moscow region, Russia). The fungus was maintained on potato-carrot agar (PCA medium: 200 g of white potato, 200 g of carrot, 20 g of agar per 1 L of medium) in Petri dishes. For the experiment, a 0.5 × 0.5 cm^3^ agar block with mycelium was transferred to a new Petri dish and incubated at +24 °C until the onset of sporulation. Conidia were washed off the colony surface with 5 mL of sterile water, and then the suspension was filtered through a sterile cloth to remove conidia fragments. The concentration of conidiospores in the stock suspension was evaluated using a Goryaev chamber; if necessary, the suspension was diluted with sterile water to the required conidia concentration.

### 2.2. Experimental Design

*F. ulmaria* seeds were surface sterilized with 70% ethanol for 5 min, followed by triple rinsing with a large volume of sterile water. The sterilized seeds were sown in a pre-steamed mixture of soil and perlite (1:2, *v*/*v*) in four plastic containers with 100 seeds in each container. For cold stratification, closed containers were placed in the dark at +8 °C for three months, whereupon they were transferred to a climate chamber at +25 °C for two weeks until the emergence of two true leaves. At this stage, plants from each container were divided into two experimental groups, pathogen-inoculated (*B. sorokiniana*) and mock-inoculated control group, by transplanting into two new containers. Thus, four experimental repeats were obtained. For inoculation, freshly prepared conidia suspension (7 mL per container at a concentration of 0.5 × 10⁶ conidia/mL) was applied to the leaves. Control seedlings were treated similarly with 7 mL of sterile water. After treatment, all plants were maintained in the climate chamber at +25 °C and a photoperiod of 16 h day/8 h night. Four samples of plant tissue for RNA isolation from the control and infected groups were taken 24 and 48 h post inoculation (hpi) or water treatment. They were denoted as control samples 1 and 3 (24 and 48 hpi, respectively) and infected samples 2 and 4 (24 and 48 hpi, respectively). For each experimental group, a pooled sample of 8–12 whole plants (200–250 mg of plant tissue) from four containers was cut into pieces and divided into two equal parts (replicates a and b), each of which was fixed in 1 mL of IntactRNA reagent (Eurogen, Moscow, Russia) in Eppendorf tubes.

### 2.3. RNA Isolation

Total RNA was isolated using Plant RNA Isolation Aid kit (Ambion, ThermoFisher, Waltham, MA, USA) according to the protocol recommended by the manufacturer. RNA concentration was determined on a Qubit fluorimeter (Invitrogen, ThermoFisher, Waltham, MA, USA), and the quality of the RNA preparations was checked on an Agilent 2100 Bioanalyzer (Agilent Technologies, Santa Clara, CA, USA). One half of each RNA sample was used for production of eight cDNA libraries for Illumina NextSeq 500 (Illumina, San Diego, CA, USA) sequencing; the remaining half was used for qRT-PCR validation.

### 2.4. Library Construction and NGS

mRNA from eight *F. ulmaria* samples 1a, 1b, 2a, 2b, 3a, 3b, 4a, and 4b was enriched using oligo (dT) beads. cDNA libraries were obtained with the TruSeq stranded mRNA library Prep kit (Illumina) according to the manufacturer’s protocol. The quality of cDNA libraries was checked with Agilent 2100 Bioanalyzer (Agilent Technologies). Illumina NextSeq 500 sequencing was carried out on the equipment of EIMB RAS “Genome” Center. For the libraries 1a, 1b, 2a and 2b 75-bp single-end reads, and for 3a, 3b, 4a, 4b, 150-bp paired-end reads were obtained. FASTQ files were produced with bcl2fastq Conversion Software v1.8.4 (Illumina).

### 2.5. Sequencing Data Analysis

Read quality was assessed using FastQC (version 0.11.9) [51]. To remove adapter sequences and low-quality reads, raw reads from each sample were trimmed with Trimmomatic software (version 0.38) using the following parameters: “ILLUMINACLIP: TruSeq3-PE.fa:2:30:10 LEADING:3 TRAILING:3 SLIDINGWINDOW:4:30 MINLEN:36” for samples 1 and 2; “ILLUMINACLIP: TruSeq3-PE.fa:2:20:10 LEADING:3 TRAILING:3 SLIDINGWINDOW:4:30” for samples 3 and 4 [52]. Trimmed reads from each sample were combined and de novo assembled using rnaSPAdes software (version 3.15.4) with default parameters [53]. Assembled transcripts were annotated using TransDecoder (version 5.5.0) software with default parameters [54]. The quality of transcriptome assembly was evaluated with QUAST (version 5.0.2) [55]. Transcriptome assembly and annotation completeness were also assessed with BUSCO (version 5.2.10) software with parameters “m prot -l viridiplantae_odb10” [56].

### 2.6. Identification of CRP and Pep Precursors in F. ulmaria Transcriptomes

The pipeline for the identification of putative CRPs was described earlier [57,58]. Peps were discovered by BLAST search using the available sequences of orthologs found in Rosaceae plants. Signal peptides were predicted with SignalP 6.0 [59]. All identified putative AMPs were tested by the CAMPR4 program to predict if they belonged to antimicrobial peptides [60]. Isoelectric point (pI) for each putative mature peptide was calculated by IPC tool [61]. The C-terminal glycosylphosphatidylinositol-anchored (GPI-anchored) signals of nsLTPs were predicted by the big-PI PPlant Predictor program [62]. All alignments were constructed using Vector NTI Advance 9 software (Invitrogen).

### 2.7. Analysis of DEGs

Differential gene expression analysis was based on read counts from infected seedlings compared to those obtained from untreated control seedlings. DEGs were identified using DESeq2 package [63]. Clean reads were mapped to the combined transcriptome assembly using BWA MEM algorithm and SAMtools [64,65]. Expression values were calculated as counts per million (CPM) mapped reads. Minimal expression threshold was defined as 0.3. DEGs were those with an expression fold change ≥1.5 (up-regulation) or ≤0.5 (down-regulation) and FDR ≤ 0.5. CRP gene expression patterns were represented by heat maps (R package gplots v3.0.1).

### 2.8. Validation of RNA-Seq Data by qRT-PCR Analysis

To validate the transcriptomic data, 10 genes were randomly selected for qRT-PCR analysis. RNA obtained by mixing RNA preparations from two replicates of the same sample in an equal ratio was used for cDNA synthesis with oligo(dT) primer and Mint cDNA synthesis kit (Evrogen) according to the manufacturer’s instructions. The list of primers used in PCR is shown in Table S1. qRT-PCR was performed using the qPCRmix-HS SYBR+HighROX kit (Eurogen) according to the manufacturer’s protocol on a DT-96 Real-Time Instrument (DNA-technology, Moscow, Russia). PCR conditions were as follows: initial denaturation step at 94 °C for 2 min followed by 40 cycles of denaturation at 94 °C for 30 s, primer annealing at 59–60 °C for 30 s, and primer extension at 72 °C for 30 s, with the final extension of 5 min at 72 °C. The EF1-α (elongation factor 1-alpha) gene of *F. ulmaria* was used as the internal control (Table S2). Each experiment was run in three technical replicates. The relative abundance of transcripts was estimated using the 2^−∆∆CT^ method [66]. The PCR amplification specificities of genes were confirmed by sequencing the PCR fragment. The results were presented as the mean ± standard deviation (SD).

## 3. Results

### 3.1. Transcriptome Sequencing

To explore the effect of *B. sorokiniana* infection on *F. ulmaria* seedlings, RNA-seq of 8 cDNA libraries obtained from infected at 24 and 48 hpi plants (samples 2 and 4 in two replicates a and b, respectively) and mock-inoculated at the same time points control plants (samples 1 and 3 in two replicates a and b, respectively) were sequenced on Illumina NextSeq 500 platform. As a result, 227,855,151 single-end raw reads were obtained from samples 1 and 2. Samples 3 and 4 produced 153,580,388 paired-end raw reads. Raw reads generated by the sequencer were preprocessed to remove adapter sequences and low-quality reads, resulting in 167,567,433 single-end trimmed reads for samples 1 and 2, and 127,920,462 paired-end reads for samples 3 and 4. Trimmed reads from the control, infected and combined libraries were de novo assembled using transcriptome assembler software rnaSPAdes [53]. Assembly statistics for the combined sample are shown in Table 1. The quality of assemblies was assessed by BUSCO [56]. The results are shown in Table 2. A small number of missing single-copy orthologs (missing BUSCOs) pointed to sufficient quality of the assembly for further analysis.

### 3.2. Identification of Precursors Encoding AMPs and Signaling Peptides

#### 3.2.1. CRPs

Analysis of *F. ulmaria* transcriptomes using the algorithm for CRP identification based on cysteine signatures revealed 80 transcripts of putative CRPs. They encoded antimicrobial and signaling peptides of the following families: defensin-like peptides (DEFLs), nsLTPs, snakins, thionins, MEG and pollen Ole e 1 peptides, and RALFs.

DEFLs

**Figure 1:**
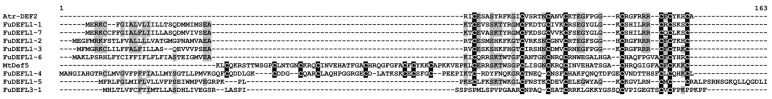
Multiple sequence alignment of *F. ulmaria* DEFL precursors, *Amaranthus tricolor* defensin Atr-DEF2 [67] and *Medicago truncatula* MtDef5 [68]. Cysteine residues are shaded black, and identical amino acids are shaded grey.

Most *F. ulmaria* DEFLs were synthesized as precursor proteins consisting of a signal peptide and a mature peptide characteristic of class 1 defensins (Figure 1). A few DEFL precursors possessed a C-terminal domain typical for class 2 defensins. *F. ulmaria* DEFLs included five classical defensins with a 8-Cys motif: C-X{4,25}-C-X{2,12}-C-X{3,4}-C-X{3,17}-C-X{4,32}-C-X-C-X{1,6}-C, one modified FuDEFL1-5 with a Cys-Cys pair, and one bidomain FuDEFL1-4 (Figure 1). In addition to 8-Cys peptides, *F. ulmaria* DEFL family included one 6-Cys DEFL3-1, which conforms to the motif: C-X{2,14}-C-X{3,5}-C-X{3,16}-C-X{4,28}-C-X-C. FuDEFLs 1-1, 1-2, 1-3, 1-6 and 1-7 were annotated as defensin-like peptides, while the remaining peptides, as hypothetical proteins (Table S2, Figure 1).

All *F. ulmaria* DEFLs showed the highest sequence similarity to Rosaceae plants’ defensins. Nevertheless, it is worth noting that the sequence identity with defensins of Rosaceae plants is not very high ranging from 40 to 84%. All but one FuDEFL1-4 of the predicted DEFLs were basic, which is characteristic of AMPs (Table S2). Correspondingly, all but one identified *F. ulmaria* DEFLs were predicted to belong to AMPs.

**Figure 2:**
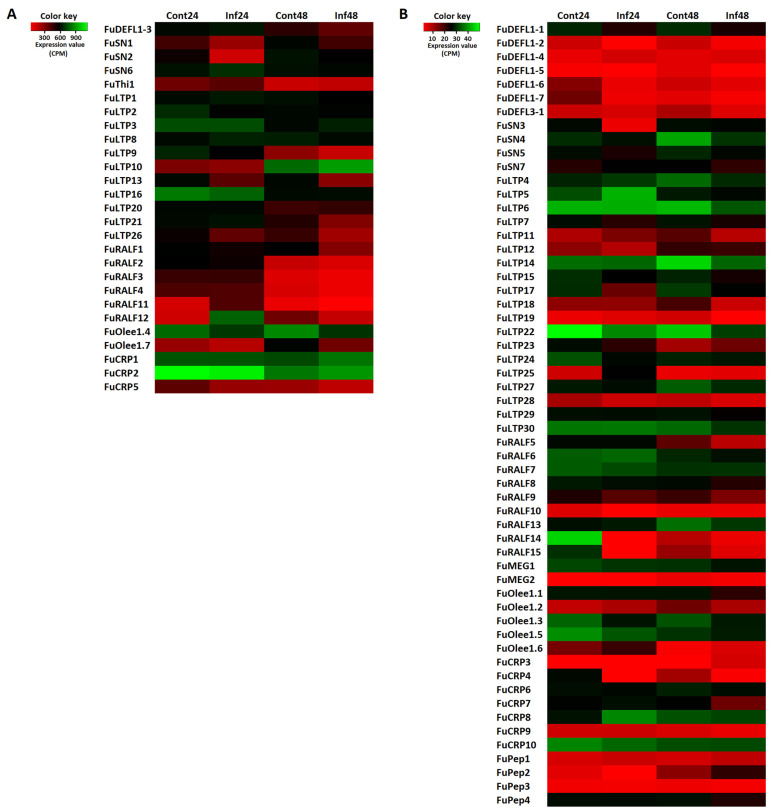
Heatmaps of differential gene expression. (**A**) Genes with expression levels above 60 CPM at least in one transcriptome. (**B**) Genes with expression levels below 60 CPM in all transcriptomes. Cont24, Cont48, Inf24 and Inf48 designate control and infected plants at 24 or 48 hpi, respectively.

Expression profiling showed that meadowsweet DEFL genes differed in expression levels, which was the highest for FuDEFL1-3 both in control and infected plants (Figure 2). The FuDEFL1-3 gene was slightly (1.4-fold) up-regulated by *B. sorokiniana* at 24 hpi; however, at the later infection stage, its expression level decreased (Figure 2, Table S3). Of the weakly expressed genes, only FuDEFL1-4 gene was up-regulated by *B. sorokiniana* infection at 24 hpi. Three DEFL genes (FuDEFL1-1, 1-6 and 1-7) were down-regulated by the fungus at 24 hpi (Figure 2, Table S3). Only FuDEFL1-1 and FuDEFL3-1 were down-regulated at 48 hpi. The expression levels of other DEFL genes were unaffected by the fungus.

Snakins

**Figure 3:**
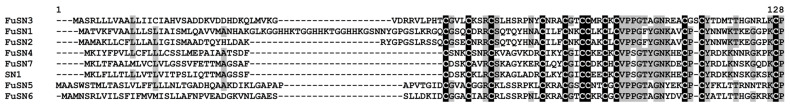
Multiple sequence alignment of *F. ulmaria* snakin precursors and *Solanum tuberosum* SN1 (Q948Z4.1). Cysteine residues are shaded black, and identical amino acids are shaded grey.

Seven snakins named FuSN1–7 were discovered in *F. ulmaria* transcriptomes (Figure 3). They were synthesized as precursor proteins consisting of a signal peptide and a mature peptide containing 12 cysteines. They showed the highest sequence similarity with gibberellin-regulated proteins or snakins of the Rosaceae plants (67–89% sequence identity) (Table S2). All snakins were basic and predicted to possess antimicrobial properties.

*F. ulmaria* snakins varied in transcription levels in different transcriptomes (Figure 2). The highly expressed FuSN6 gene was the only snakin gene up-regulated by *B. sorokiniana* infection at 24 hpi (Table S3). Two snakin genes, FuSN2 and 3, were down-regulated at 24 hpi and FuSN4 at 48 hpi. Comparison of snakin gene expression at 24 and 48 hpi shows that the expression level of four snakin genes, FuSN1–4, increased at the later infection stage (Table S3).

nsLTPs

**Figure 4:**
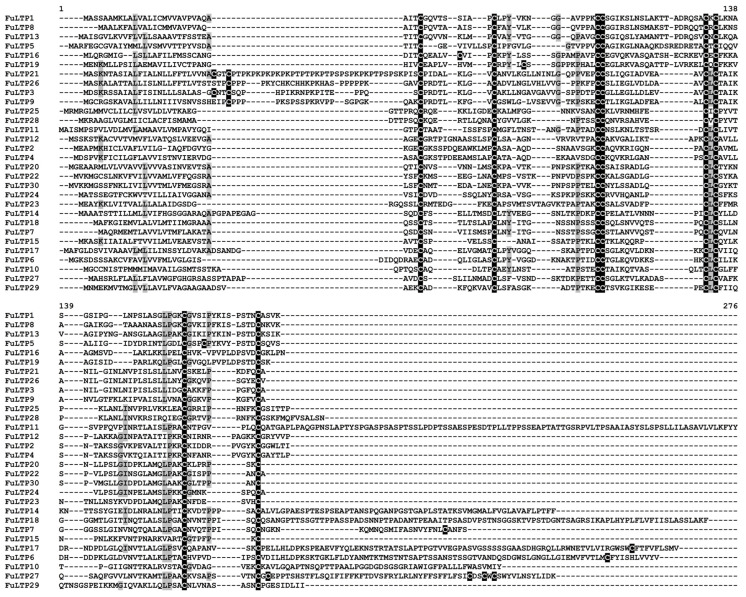
Multiple sequence alignment of *F. ulmaria* nsLTP precursors. Cysteine residues are shaded black, and identical amino acids are shaded grey.

Thirty nsLTP transcripts were identified in *F. ulmaria* transcriptomes (Figure 4). The predicted peptides exhibited sequence similarity with Rosaceae nsLTPs (Table S2). Sequence identity varied from 55 to 89%. They included both basic and acidic polypeptides with or without a GPI-anchor (Table S2). Eighteen peptides were predicted to have antimicrobial activity; the remaining peptides were classified as non-AMPs.

Eleven *F. ulmaria* nsLTP genes (FuLTP1, 2, 3, 8, 9, 10, 13, 16, 20, 21 and 26) were highly expressed at least in one transcriptome (Figure 2). Of them, only the FuLTP8 gene was up-regulated upon infection at 24 hpi and FuLTP3 at 48 hpi. The transcript level of FuLTP10 increased from 24 to 48 hpi (Table S3). Among the weakly expressed genes, FuLTP5, 11, 19 and 25, were up-regulated by *B. sorokininana* at 24 hpi, and FuLTP23 at 48 hpi (Table S3). Progress of the infection from 24 to 48 h was accompanied by up-regulation of three nsLTP genes (FuLTP12, 17, 27). Six nsLTP genes (FuLTP2, 9, 13, 15, 17, 24) were down-regulated by the fungus at 24 hpi, and six genes (FuLTP11, 13, 15, 17, 18 and 22) were down-regulated at 48 hpi (Table S3).

Thionin-Like Peptides

**Figure 5:**
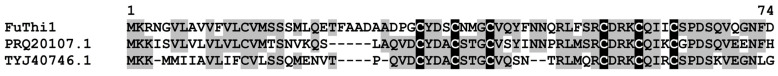
Multiple sequence alignment of *F. ulmaria* thionin precursor and homologs from *Rosa chinensis* (PRQ20107.1) and *Gossypium mustelinum* (TYJ40746.1). Cysteine residues are shaded black, and identical amino acids are shaded grey.

Only one thionin-like peptide FuThi1 was predicted in *F. ulmaria* transcriptomes (Figure 5). It lacked the typical thionin sequence, although it possessed a C-terminal domain of the thionin precursor with six conserved cysteine residues. It showed a sequence identity of 58% to a hypothetical protein PRQ20107.1 of *Rosa chinensis*. The predicted peptide was acidic (had pI value of 4.007) and devoid of antimicrobial properties (Table S2). The expression level of the FuThi1 gene decreased by 48 hpi (Figure 2, Table S3).

MEG Peptides

**Figure 6:**
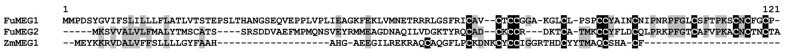
Multiple sequence alignment of *F. ulmaria* MEG precursors and *Zea mays* MEG1 (XP_008652138.1). Cysteine residues are shaded black, and identical amino acids are shaded grey.

Two MEG peptide transcripts encoding FuMEG1 and FuMEG2 were detected in *F. ulmaria* transcriptomes (Figure 6). They were 74% and 56% identical to the hypothetical protein ONI05184.1 of *Prunus persica* and uncharacterized protein XP_024189479.1 of *R. chinensis*, respectively (Table S2).

The expression level of FuMEG1 gene was higher than that of FuMEG2 gene in transcriptomes of control and infected plants at both time points (Figure 2). However, no significant changes in expression levels of both MEG genes were observed upon *B. sorokiniana* infection (Table S3).

Ole e 1 Peptides

**Figure 7:**
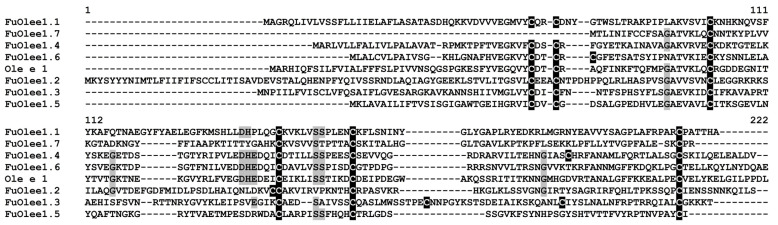
Multiple sequence alignment of *F. ulmaria* Ole e 1 precursors and *Olea europaea* Ole e 1-like allergens (XP_022872526.1). Cysteine residues are shaded black, and identical amino acids are shaded grey.

Seven Ole e 1 peptides were predicted in *F. ulmaria* transcriptomes (Figure 7). They showed the highest sequence similarity (from 70 to 88%) to the uncharacterized proteins of Rosaceae plants. The predicted peptides were either basic or acidic, and all of them were presumably AMPs (Table S2).

Only FuOlee1.4 was highly expressed in all four transcriptomes; however, its expression level decreased at 48 hpi (Figure 2). The transcript level of FuOlee1.7 increased from 24 to 48 hpi (Table S3). Among weakly expressed genes, the expression level of FuOlee1.1 decreased at 48 hpi, while that of FuOlee1.3 decreased at 24 hpi and 48 hpi (Table S3).

RALFs

**Figure 8:**
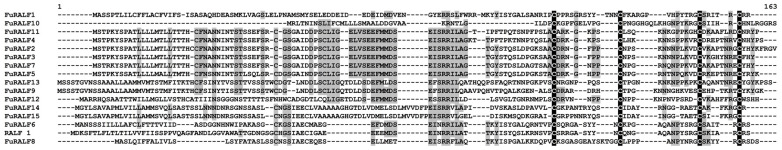
Multiple sequence alignment of *F. ulmaria* RALF precursors and RALF 1 from *Arabidopsis thaliana* (NP_171789.1). Cysteine residues are shaded black, and identical amino acids are shaded grey.

Fifteen RALF precursors were predicted in *F. ulmaria* transcriptomes (Figure 8). Most of them showed from 44 to 85% sequence identity to RALF-like or hypothetical proteins of Rosaceae plants, while FuRALF14 and 15 showed sequence similarity to the hypothetical protein COLO4_37988 of *Corchorus olitoris* (Malvaceae) (30% and 35%, respectively) (Table S2). They also possessed a dibasic site assumed to be necessary for the proteolytic cleavage of RALF precursors.

Expression profiling showed that six FuRALF genes encoding FuRALFs 1–4, 11 and 12 were strongly expressed in *F. ulmaria* transcriptomes (Figure 2). FuRALF11 and 12 were up-regulated at 24 hpi, while six RALF genes including FuRALF11 and 12 were down-regulated at 48 hpi (Table S3). Progression of infection from 24 to 48 h is associated with down-regulation of seven FuRALF genes (Table S3). Only FuRALF13 was up-regulated.

#### 3.2.2. CRPs with Novel Cysteine Motifs

4-Cys Peptides FuCRP5-10

**Figure 9:**
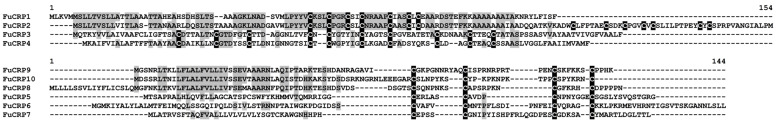
Multiple sequence alignment of *F. ulmaria* CRP precursors with novel cysteine motifs. Cysteine residues are shaded black, and identical amino acids are shaded grey.

A new family of 4-Cys containing peptides was discovered in *F. ulmaria* transcriptomes (Figure 9, Table S2). They were produced as precursor proteins containing a signal peptide and a mature peptide domain. The cysteine motifs of the mature peptides differed from that of α-hairpinins and 4-Cys DEFLs (Figure 9). This new family included six peptides denoted FuCRP5–10. Three of them, FuCRP5–7, had no BLAST hits, while FuCRP8 and FuCRP9 showed sequence similarity to the hypothetical proteins KAG2717713.1 and KAG2714216.1 of *Carya illinoinensis* (Juglandaceae) (41% and 36%, respectively) (Table S2). FuCRP10 displayed 37% sequence identity with the hypothetical protein GBA52_014471 of *Prunus armenica*.

Only the FuCRP8 gene was activated by the fungus at 24 hpi. The expression level of FuCRP7 and FuCRP9 decreased from 24 to 48 hpi (Table S3). All other genes were insensitive to the infection.

Other CRPs

In addition to 4-Cys containing peptides, four peptides with three novel cysteine motifs were discovered in *F. ulmaria* transcriptomes (Figure 9, Table S2). Similar to 4-Cys FuCRPs, they were produced as precursor proteins containing a signal peptide and a mature peptide. Two mature peptides, FuCRP1 and 2, possessed 7 and 14 cysteine residues, respectively; FuCRP3 and 4 possessed 10 cysteine residues (Figure 9). All peptides had no BLAST hits, and so are novel genes (Table S2).

Of note, two transcripts encoding FuCRP1 and 2 were highly expressed in *F. ulmaria* transcriptomes, although their expression level was unaffected by *B. sorokiniana* infection (Figure 2).

#### 3.2.3. Peps

**Figure 10:**
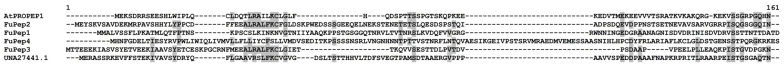
Multiple sequence alignment of *F. ulmaria* Pep precursors, Pep2 precursor from *Prunus persica* (UNA27441.1) and *A. thaliana* PROPEP1 [69]. Identical amino acids are shaded grey.

Four transcripts encoding Pep precursors (FuPep1–4) were discovered in *F. ulmaria* transcriptomes (Figure 10). FuPep3 showed similarity to the Pep precursor from *P. persica* var. *nucipersica* UNA27441.1 (46% identity). The remaining sequences displayed similarity to the uncharacterized proteins of *Fragaria vesca* (FuPep4 with 78% identity) and *R. chinensis* (FuPep1 and 2 with 56–57% identity) (Table S2).

Of all FuPeps, the expression level of FuPep4 was the highest in all transcriptomes; however, it did not change after infection with the fungus (Figure 2). The FuPep2 gene appeared to be responsive to infection. It was up-regulated at 48 hpi (Table S3). FuPep1 and 3 were insensitive to infection.

### 3.3. Validation of RNA-Seq Data by qRT-PCR Analysis

**Figure 11:**
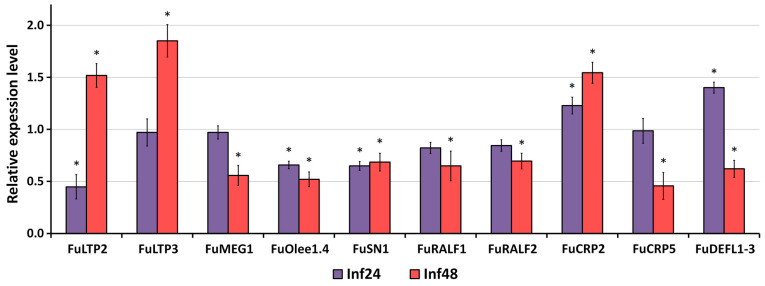
qRT-PCR validation of expression levels for selected *F. ulmaria* CRP genes. Relative expression values were normalized using the EF1-α gene as internal control and standardized relative to the control values. Analyses were accomplished in triplicate. Bars represent mean ± standard deviation (SD). Asterisks indicate significant differences between infected and control plants (Student’s *t*-test, *p* < 0.05; *n* = 3).

To confirm the gene expression profiles obtained by RNA-seq, 10 genes encoding CRPs belonging to different families were selected for qRT-PCR. The EF1-α gene of *F. ulmaria* was used as an internal control. The qRT-PCR results confirmed that the expression patterns of all selected genes were consistent with the RNA-seq data, except for the FuLTP2 and FuCRP2 genes (Figure 11).

## 4. Discussion

Medicinal plants represent an inexhaustible source of valuable biologically active compounds. *F. ulmaria* is no exception in this regard, the pharmacological properties of which are used in the treatment of various disorders, including infectious diseases [70,71]. Despite the widespread use of meadowsweet extracts in folk medicine, the molecular components implicated in this or that activity in this species are poorly understood. Studies have linked important biological activities of *F. ulmaria* predominantly to secondary metabolites [72,73,74,75]. Salicylic acid, the metabolite of salicylic alcohol derivatives present in *F. ulmaria*, is responsible in part for the pharmacological activity of this plant [76]. At the same time, defense proteins and peptides, which are among the most important participants of the defense arsenal of all plants, have not been studied in *F. ulmaria*.

**Figure 12:**
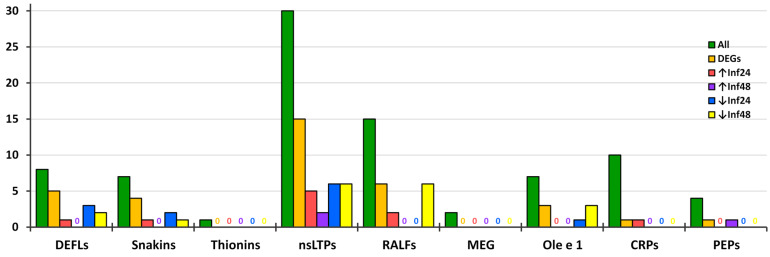
Family distribution of identified *F. ulmaria* peptide genes, DEGs, up-regulated (↑) and down-regulated (↓) genes in infected plants at 24 and 48 hpi compared to control.

Earlier, we showed that *F. ulmaria* extracts enriched in polypeptide-based molecules efficiently suppress the growth of pathogenic microorganisms causing diseases in plants and humans [49]. In this work, to identify the whole complement of peptides produced in meadowsweet in response to infection with the pathogenic fungus *B. sorokiniana*, we used transcriptomic analysis. We have applied our previously developed algorithm for searching for antimicrobial and signaling peptides in transcriptomic data by cysteine signatures, which proved successful for other plant species [57,58,77,78]. As a result, in *F. ulmaria*, we identified as many as 84 AMPs and signaling peptides, which belong to known, as well as new peptide families (Table S2). The abundance of *F. ulmaria* peptides in each family is shown in Figure 12. nsLTPs represent the most numerous family followed by RALFs, CRPs with novel cysteine motifs, defensins and snakins. BLAST search in NCBI databases showed that all discovered peptides were new: none of them were 100% identical to a known protein. Several peptides had no BLAST hit, and thus were encoded by novel, previously uncharacterized genes. In most instances, the discovered peptides showed the highest sequence identity to the peptides from Rosaceae plants. In single cases, the highest similarity was also observed with the peptides from other plant families pointing to a wider distribution of a peptide beyond the Rosaceae family. Comparison of gene expression patterns of AMPs and signaling peptides at 24 and 48 hpi demonstrates clear-cut differences, which reflect the dynamics of the defense response to *B. sorokiniana* infection.

Of the defensins, the most ancient and ubiquitous AMP family, five family members with the classical 8-Cys motif were found in *F. ulmaria*. FuDEFL1-3 is the only defensin, which is highly expressed in all *F. ulmaria* transcriptomes, and it is slightly up-regulated upon infection. We can assume that, together with other 15 constitutively and highly expressed AMP genes (FuSN1, FuThi1, FuLTP1, FuLTP10, FuLTP16, FuLTP20, FuLTP21, FuLTP26, FuRALF2–4, FuOlee1.7, FuCRP1, FuCRP2, FuCRP5) (Figure 2), the FuDEFL1-3 gene provides basal defense against attacking pathogens. One discovered *F. ulmaria* defensin FuDEFL1-5 has a modified Cys signature with a Cys-Cys pair. We believe that it originated from the “normal” *F. ulmaria* defensin due to mutations. Sequence similarity with other meadowsweet defensins supports this hypothesis. Another interesting finding is the discovery of a bidomain defensin FuDEFL1-4. To the best of our knowledge, only one bidomain defensin MtDef5 from *Medicago truncatula* has been described so far in plants [68]. Sequence analysis of discovered defensins allowed us to draw conclusions about the antimicrobial activity of some *F. ulmaria* defensins. FuDEFL1-2 and FuDEFL1-3 harbor a RGFRRR sequence in the γ-core motif, which is responsible for antimicrobial activity of plant defensins [79]. This small basic peptide discovered in defensins of taxonomically diverse plants [80], alone displays antimicrobial activity, as was clearly shown for *M. truncatula* defensin MtDef4 [81]. The γ-core motifs possessing this sequence also exhibit antimicrobial properties [82]. In our previous work, we showed that the γ-cores of wheat and tomato defensins with RGFRRR sequence display strong antimicrobial activity against a broad spectrum of plant and human fungal and bacterial pathogens [83,84]. For example, the IC_50_ for pathogen inhibition by the wheat DEFL1-16 γ-core with RGFRRR sequence was 4.4 µM for *Cryptococcus neoformans*, 14.6 µM for *Candida albicans*, 12.1 µM for *Fusarium oxysporum*, 20.7 µM for *Fusarium culmorum* and 14.6 µM for *Clavibacter michiganensis* [84]. From these data on RGFRRR-sequence-containing defensins we can conclude that *F. ulmaria* FuDEFL1-2 and FuDEFL1-3 are indeed antimicrobial peptides.

In addition to 8-Cys defensins, in *F. ulmaria* transcriptome, we also discovered a 6-Cys DEFL, FuDEFL3-1. The FuDEFL3-1 molecule is basic, pointing to its antimicrobial potential. An intriguing finding is that all but one *F. ulmaria* defensin DEGs were down-regulated by *B. sorokiniana* infection. Only the negatively charged bidomain FuDEFL1-4 was up-regulated (Table 3). Accordingly, *B. sorokiniana* suppresses most defensin production in *F. ulmaria*.

Similar to defensins, only one of seven discovered in *F. ulmaria* transcriptomes snakin genes encoding FuSN6 was up-regulated by *B sorokiniana* (Table 3). Snakin FuSN6 is predicted to belong to AMPs. Furthermore, it is nearly identical in the γ-core region to tomato snakin SlSN2, whose potent antimicrobial activity was shown in our previous work [83]. Therefore, it also displays antimicrobial activity. Two snakin genes were down-regulated by the infection at 24 hpi and one gene at 48 hpi, the remaining genes were insensitive to the fungus.

nsLTPs constitute the most abundant AMP family in *F. ulmaria* transcriptomes represented by 30 family members (Figure 4 and Figure 12). Eleven nsLTPs were highly expressed at least in one transcriptome (Figure 2). Only five nsLTPs were up-regulated at 24 hpi, and two at 48 hpi; six peptides were down-regulated at 24 hpi and six, at 48 hpi (Table 3). The expression level of other nsLTP genes did not change significantly upon the fungal infection.

Minor CRP families in *F. ulmaria* were represented by one thionin-like peptide FuThi1, two MEG peptides FuMEG1 and FuMEG2 and seven Ole e 1 peptides. Of these CRP genes, none were up-regulated by *B. sorokiniana* infection (Figure 12, Table S3). The vast majority of genes were irresponsive to the fungus, and only three Ole e 1 genes were down-regulated (Table 3).

Of special interest is the discovery of *F. ulmaria* transcriptomes of 10 CRP genes with novel cysteine motifs. Among them, peptides with 4, 7, 10, and 14 cysteines were detected. Only the 4-Cys FuCRP8 gene was up-regulated by *B. sorokiniana* infection, suggesting its direct participation in defense either as an antimicrobial or as a modulator of the immune response (Table 3 and Table S3). Further functional studies of this and other novel CRPs are necessary.

Transcriptome analysis revealed two families of signaling peptides in *F. ulmaria:* RALFs and PEPs. RALF (RAPID ALKALINIZATION FACTOR) peptides cause rapid pH increase in tobacco cell cultures and belong to CRPs produced from the C-terminal region of a preproprotein [85]. RALFs are involved in regulation of plant growth and development, including root cell elongation, pollen tube growth, and nodulation [86,87,88,89,90,91,92]. However, the accumulating data indicate that RALF-mediated signaling also participates in plant-microbe interactions modulating PTI (Pathogen-Triggered Immunity) [93]. We discovered 15 RALF-like peptides in *F. ulmaria* transcriptomes. For comparison, *Arabidopsis* genome carries more than 30 RALFs, and they were shown to have different functions serving as negative or positive regulators in plant immunity [92]. Furthermore, only AtRALFs with the dibasic proteolytic processing site RR were demonstrated to be involved in immunity regulation [93,94,95]. Similar to *Arabidopsis*, in *F. ulmaria* transcriptome we discovered RALFs with and without this processing site. However, all differentially expressed FuRALF genes possessed a dibasic proteolytic processing site; therefore, they are likely to participate in immune response to infection. Two FuRALF genes were up-regulated at 24 hpi, while six FuRALF genes were down-regulated at 48 hpi (Table 3). Taking into consideration the above-mentioned data concerning *Arabidopsis* RALFs, it is tempting to speculate that *F. ulmaria* RALFs fulfill diverse functions, acting both as positive and negative immune regulators. The suggestion that two up-regulated FuRALF genes suppress plant growth to activate defense reactions cannot be excluded either.

Another group of signaling peptides named Peps (Plant elicitor peptides), which usually contain from 23 to 36 amino acid residues and are also derived from the C terminus of the precursor proteins PROPEPs [96], was discovered in *F. ulmaria* transcriptomes. Mature Peps of different plant species contain family-specific motifs [97,98]. Peps act as DAMPs (Damage-Associated Molecular Patterns) eliciting plant PTI responses, such as the production of reactive oxygen species, phosphorylation of MAP kinases, changes in defense gene expression, and deposition of callose in cell walls, ultimately resulting in resistance against diverse pathogens [69,96,99,100,101,102]. Peps were also shown to increase abiotic stress tolerance in plants [103].

Four Pep precursors FuPeps 1–4 were discovered in *F. ulmaria* transcriptomes. Only the FuPep2 gene was responsive to *B. sorokiniana* infection: it was up-regulated at 48 hpi (Table 3 and Table S3). We can suggest that up-regulation of the FuPep2 gene at 48 hpi leads to activation of defense reactions in *F. ulmaria*.

**Figure 13:**
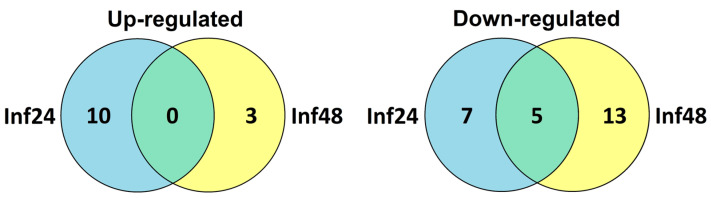
Venn diagram showing the number of DEGs specifically up- or down-regulated in infected seedlings at 24 hpi and 48 hpi as well as similarly expressed genes in both transcriptomes. For up-regulated genes, expression fold change was ≥1.5, for down-regulated, it was ≤0.5.

Thus, our results show that 42% of *F. ulmaria* CRP and Pep genes are sensitive to *B. sorokiniana* infection. Only a limited number of DEGs were up-regulated by the infection (Table 3, Figure 13). These genes are supposed to directly participate in inhibition of pathogen growth. Snakin FuSN6 displays antimicrobial activity. FuDEFL1-4 is a two-domain acidic peptide. Although many AMPs are cationic, some are anionic [104]. So, we cannot rule out the antimicrobial properties of FuDEFL1-4. However, this suggestion needs to be proved experimentally, for example by testing antimicrobial activity of its γ-core peptide. All up-regulated nsLTPs were predicted to possess antimicrobial properties. However, direct antimicrobial assays of isolated peptides, or overexpression of their genes in transgenic plants is necessary to prove their antimicrobial activity.

Transcription profiling showed that most DEGs in *F. ulmaria* were down-regulated by the fungus, suggesting suppression of CRP production by the fungus. Similar results were obtained in our studies of tomato and *Stellaria media* plants infected by *F. oxysporum* [58,77].

## 5. Conclusions

In summary, transcriptomes of healthy and *B. sorokiniana*-infected *F. ulmaria* plants were sequenced on the Illumina NextSeq 500 platform and de novo assembled. Using an earlier developed pipeline, a total of 84 genes encoding novel AMPs and signaling peptides were predicted in *F. ulmaria* transcriptomes. The discovered transcripts belong to gene families which vary widely in the number of constituent members. Transcriptional profiling of the CRP and Pep genes in healthy and infected *F. ulmaria* seedlings disclosed complex and diversified expression patterns, including both up- and down-regulated genes, which provides evidence for the diversification of functions among family members. Our results show that only some peptide genes within each family were responsive to *B. sorokiniana* infection. Constitutively expressed peptide genes might provide basal resistance to pathogens. Among DEGs, the down-regulated genes prevailed, and only a few were up-regulated by the infection. The up-regulated CRP and Pep genes are likely to directly participate in triggering defense response to the *B. sorokiniana* infection. In contrast, down-regulation of the CRP genes may reflect suppression of the immune response by the fungus or its activation by inhibiting negative regulators of immunity (e.g., RALFs). Suppression of the CRP genes associated with developmental processes as a means to redirect plant resources to defense cannot be excluded either. In any case, the role of different AMPs and signaling peptides in interactions of *F. ulmaria* plants with *B. sorokniana* needs to be further uncovered experimentally. After extensive functional studies, these new molecules will contribute to a pool of novel antimicrobials and resistance inducers derived from wild plants for practical applications.

## Figures and Tables

**Table 1:** Assembly statistics for the combined sample (1a, 1b, 2a, 2b, 3a, 3b, 4a, 4b).

Parameter	Number of Raw Assembled Contigs	Number of CDS *
Number of sequences	158,615	46,063
N50, bp	1365	1221
Median length, bp	297	699
Average length, bp	687	933
Maximal length, bp	16,604	14,925

* Coding DNA sequences.

**Table 2:** Quality evaluation of transcriptome assemblies with BUSCO.

Assembly	Complete BUSCOs (%)	Complete and Single-Copy BUSCOs (%)	Complete and Duplicated BUSCOs (%)	Fragmented BUSCOs (%)	Missing BUSCOs (%)
1a,1b,2a,2b	237 (55.1)	209 (48.6)	28 (6.5)	116 (27.0)	77 (17.9)
3a,3b,4a,4b	383 (89.1)	335 (77.9)	48 (11.2)	29 (6.7)	18 (4.2)
1a,1b,2a,2b, 3a,3b,4a,4b	351 (81.6)	264 (61.4)	87 (20.2)	55 (12.8)	24 (5.6)

**Table 3:** Up- and down-regulated peptide genes in infected *F. ulmaria* plants *.

Up-Regulated		Down-Regulated	
at 24 hpi	at 48 hpi	at 24 hpi	at 48 hpi
FuDEFL1-4		FuDEFL1-1	FuDEFL1-1
		FuDEFL1-6	FuDEFL3-1
		FuDEFL1-7	
FuSN6		FuSN2	FuSN4
		FuSN3	
FuLTP5	FuLTP3	FuLTP2	FuLTP11
FuLTP8	FuLTP23	FuLTP9	FuLTP13
FuLTP11		FuLTP13	FuLTP15
FuLTP19		FuLTP15	FuLTP17
FuLTP25		FuLTP17	FuLTP18
		FuLTP24	FuLTP22
FuRALF11			FuRALF1
FuRALF12			FuRALF5
			FuRALF11
			FuRALF12
			FuRALF14
			FuRALF15
		FuOlee1.3	FuOlee1.1
			FuOlee1.3
			FuOlee1.4
FuCRP8			
	FuPEP2		

* Up-regulated genes are those with an expression fold change ≥1.5, down-regulated genes are those with an expression fold change ≤0.5.

## Data Availability

Sequencing data were deposited in NCBI at BioProject accession number PRJNA1047088.

## Supplementary Materials

The following supporting information can be downloaded at: https://www.mdpi.com/article/10.3390/jof10040258/s1, Table S1: List of primers for qRT-PCR validation; Table S2: *F. ulmaria* CRPs, Peps and EF1-α [59,60,61,62]; Table S3: Expression patterns of *F. ulmaria* CRP and Pep genes.
