# Supplementary material for: Transcriptome-Wide Identification and Expression Analysis of Genes Encoding Defense-Related Peptides of Filipendula ulmaria in Response to Bipolaris sorokiniana Infection
Source: J Fungi (Basel). 2024 Mar 28;10(4):258. doi: 10.3390/jof10040258 (PMC11050963; doi:10.3390/jof10040258)
Supplement: Supplementary file 1 [file jof-10-00258-s001.zip › Table S1.pdf]

Table S1. List of primers for qRT-PCR validation.

№	Gene	Pr_dir 5' –> 3'	Pr_rev 5' –> 3'	Length (bp)	T primer annealing (°C)
1.	*FuLTP2*	CAAATGCCGCAGTTTCTGGT	ACACCCAGCTTGCACAGTAT	220	60
2.	*FuLTP3*	TGCCCTAGGGATACCCTGAA	CTTTGATGGCGGTGCAAAGA	157	59
3.	*FuMEG1*	TGAGCAGGTAGAGCCACCA	ATGAGTTAGGGACATCCAAAGC	252	59
4.	*FuOlee1.4*	GACTCTTGCCGCTTCGGATA	GGTCACGTCCCTGAACAACT	238	60
5.	*FuSN1*	CAAGACTGGAGGTCATCACA	TTGCCATAGAAGCCAGGAGG	180	60
6.	*FuRALF1*	CATGAAGTTAGTGGCCGGGA	CGAGAGCGCACCGTATGAAA	175	60
7.	*FuRALF2*	GAGTTCTCTCGGTGTGGCAG	GATTGGTAGGGGCCTTGACG	244	59
8.	*FuCRP2*	GGTGCAGTGTGGCTTCCATA	TCAGCAGTCGGAAAAAGGCA	215	59
9.	*FuCRP5*	CCATCTGGTGACGGGCTAAG	CGGCGAGGAGGAAAACTTGA	145	60
10.	*FuDEFL1-3*	GCCCTCTTCATTCTCTTGGCT	AGCCTTCATTGCGACAGACC	136	60
11.	*FuEF1-α*	TCATCATGAACCACCCTGGC	ACTTGGGCTCCTTCTCAAGC	144	59–60
